# Enzyme-Free Monitoring of Glucose Using Molecularly Imprinted Polymers and Gold Nanoparticles

**DOI:** 10.3390/bios15080537

**Published:** 2025-08-15

**Authors:** Ana Rita Aires Cardoso, Pedro Miguel Cândido Barquinha, Maria Goreti Ferreira Sales

**Affiliations:** 1 BioMark@UC/CEMMPRE (Centre for Mechanical Engineering, Materials and Processes)-ARISE (Advanced Production and Intelligent Systems), Faculty of Sciences and Technology, Department of Chemical Engineering, University of Coimbra, 3030-790 Coimbra, Portugal; uc45752@uc.pt; 2 CENIMAT|i3N, Department of Materials Science, School of Science and Technology, NOVA University Lisbon and CEMOP/UNINOVA, Campus de Caparica, 2829-516 Caparica, Portugal; pmcb@fct.unl.pt

**Keywords:** glucose, gold nanoparticles, molecularly imprinted polymer, carboxylated pyrrol, electrochemical biosensor

## Abstract

This work describes a non-enzymatic electrochemical glucose biosensor combining for the first time molecularly imprinted polymers (MIPs) for glucose concentration and gold nanoparticles (AuNPs) on screen-printed carbon electrodes (SPEs), where both MIPs and AuNPs were assembled in situ. Electrochemical impedance spectroscopy (EIS) was used to evaluate the analytical performance of the sensor, which has a linear range between 1.0 µM and 1.0 mM when standard solutions are prepared in buffer. Direct measurement of glucose was performed by chronoamperometry, measuring the oxidation current generated during direct glucose oxidation. The selectivity was tested against ascorbic acid and the results confirmed a selective discrimination of the electrode for glucose. Overall, the work presented here represents a promising tool for tracking glucose levels in serum. The use of glucose MIP on the electrode surface allows the concentration of glucose, resulting in lower detection limits, and the use of AuNPs reduces the potential required for the oxidation of glucose, which increases selectivity. In addition, this possible combination of two analytical measurements following different theoretical concepts can contribute to the accuracy of the analytical measurements. This combination can also be extended to other biomolecules that can be electrochemically oxidised at lower potentials.

## 1. Introduction

Diabetes mellitus is a chronic metabolic disease that is a major public health problem and its impact on the global population is expected to increase over time [1]. It is estimated that up to 642 million people will be affected by 2040 [2,3]. The most common cause is the inability of the pancreas to absorb the enormous fluctuations in blood glucose levels. This leads to numerous complications such as retinopathy leading to blindness, nephropathy leading to kidney failure, damage to the peripheral nerves with an increased risk of limb ulcers (foot), amputation, cardiovascular disease, or even cancer [4,5]. To prevent these complications, blood glucose levels must be controlled, which requires regular/continuous monitoring of blood glucose levels [6]. As this is a daily routine event, it is important that patients do this themselves with an inexpensive, easy-to-use, and less invasive procedure.

A series of glucose biosensors were developed for this purpose [7]. The first electrode for monitoring glucose was proposed by Clark and Lyons in 1962 [8]. They are mostly based on the selective oxidation of glucose by glucose oxidase to later detect the decrease of a reactant or the appearance of a (side)product (Equation (1)). Regardless of whether the monitoring is continuous or discrete, invasive or non-invasive, enzyme-based detection in common glucose metres used by patients is performed by electrochemical measurements [9]. However, optical measurements have also been described in the literature [10] since the first colourimetric sensor was developed in 1965 [11], based on the detection of hydrogen peroxide produced during the oxidation of glucose by glucose oxidase (GOx).

(1) $$Glucose+O_{2}+H_{2}O\overset{GOx}{\to }Gluconicacid+H_{2}O_{2}$$

Over time, enzyme-based glucose biosensors have become the most important glucose measurement devices on the market and have gone through several generations: (1) the first generation, which uses oxygen as a co-substrate of glucose oxidase and also relies on the formation and release of H_2_O_2_; (2) the second generation, which replaces oxygen with redox mediators that transfer electrons from the enzymatic reaction; (3) and the third generation, which involves direct transfer of electrons between enzyme and electrode in the absence of the mediator [12].

However, biosensors based on enzymes have specific disadvantages related to the change in their activity under different conditions, such as temperature, pH, storage, and specific inhibitors [13]. Biosensors without enzymes can improve reliability by eliminating the drift caused by enzymatic degradation [14]. These include several studies using metal catalysts such as Au, Pt, Cu, Ni, or single polycrystalline metals (Au, Pt, and Bo-iron doped diamond) in solution, or in the form of nanoparticles (NPs) [15], also called nanoenzymes. Nevertheless, these sensors, which use only non-biological catalytic materials, are able to co-oxidise compounds such as ascorbic acid (AA), uric acid (UA), and paracetamol (APH) in addition to glucose [16].

An important approach to mitigate the limitations of enzymes is the combination of molecularly imprinted polymers (MIPs) [17] with enzyme-free catalytic approaches such as nanoenzymes. The MIP materials provide selectivity in the final analysis by selectively accumulating glucose on the surface of the electrode, while the enzyme-free catalytic approaches provide a sensitive improvement in the analytical response obtained. This gain in sensitivity can be even greater if conductive polymers are used to prepare the MIP materials when an electrical reading is retrieved from the sensor system. The use of gold nanoparticles (AuNPs) for this purpose offers the additional advantages of good biocompatibility and unique photoelectron performance [18].

There are several approaches that utilise MIP materials for glucose sensing [19,20], some of which include metal nanoparticles as catalytic elements.

A critical comparison of the analytical performance of the proposed MIP-based glucose sensor with previously reported systems (summarised in Table 1) reveals several strengths. The present sensor achieves a limit of detection (LOD) of 0.15 µM with a linear response range of 1.0 µM to 1.0 mM and exhibits high reproducibility as shown by relative standard deviations (RSD) between 1.37% and 3.2% for independent measurements. These results are competitive when compared to those of Kim et al. (2017) [21], who reported an LOD of 0.19 µM using an MIP sensor on polythiophene-derivative modified AuNPs/SPCE, although their approach involved more complex synthesis and surface chemistry. Similarly, Diouf et al. (2019) [22] achieved an LOD of approximately 3.3 µM with a narrower dynamic range and lower detection sensitivity. Cho et al. (2018) [15] reported a wider linear range (1.0 µM to 25.0 mM), but at the cost of a higher LOD (0.65 µM) and more complicated bimetallic nanostructures.

The novelty of our sensor lies in the electrochemical in situ production of both the AuNPs and the MIP directly on low-cost screen-printed electrodes (SPEs). This approach simplifies the entire development process, eliminates the need for external reducing or binding agents, and improves the uniformity of the functional materials. In addition, the combination of two electrochemical detection modes—electrochemical impedance spectroscopy (EIS) and chronoamperometry, offers flexible detection strategies, with EIS providing highly sensitive probe-based measurements and chronoamperometry enabling direct and rapid glucose detection. Taken together, these features not only demonstrate analytical performance that equals or exceeds several modern systems, but also provide a scalable, cost-effective, and user-friendly platform for glucose determination in real samples.

This work describes the production of an MIP material for glucose obtained by in situ electropolymerisation of pyrrole (Py). This is done on a carbon support of screen-printed electrodes containing AuNPs prepared in situ by suitable reduction of gold salt. The conditions for carrying out these electrochemical processes were optimised with respect to the presence or absence of AuNPs on the electrode surface during pyrrole-2-carboxylic acid (Py-COOH)-MIP production. The best device was characterised in terms of its analytical properties, followed by its application in the analysis of human serum.

## 2. Experimental Section

### 2.1. Reagents and Solutions

High purity Milli-Q laboratory grade water (conductivity < 0.1 µS/cm) was used in this work. Chemical reagents used include potassium ferricyanide (K_3_[Fe(CN)_6_]), potassium ferrocyanide (K_4_[Fe(CN)_6_]) trihydrate, and phosphate buffer salt solution (PBS), purchased from Riedel-de Haeen; pyrrole (Py) and pyrrole-2-carboxylic acid 99% (Py-COOH), purchased from Alfa Aesar; tetrachloroauric acid (HAuCl_4_·H_2_O), sulfuric acid (H_2_SO_4_), and glucose, purchased from Sigma Aldrich; ascorbic acid (AA), purchased from Fluka. The human serum was purchased from Cormay (R).

All solutions were prepared in ultrapure water. An amount of 0.05 M H_2_SO_4_ was used for electrochemical cleaning of the carbon devices. A 0.1 mg/mL HAuCl_4_ solution was prepared in ultrapure water.

The MIP solution contained 1.0 mM of Glucose, 1.0 mM of Pyrrol-2-Carboxylic acid (Py-COOH), and 1.0 mM Pyrrol (Py) prepared in 0.10M PBS (pH 7.4). The Non-Imprinted Polymer (NIP) solution contained 1.0 mM Py-COOH and 1.0 mM of Pyrrole prepared in 0.10M PBS (pH 7.4). The standard solutions of glucose were prepared in 0.10 M PBS (pH 7.4). The changes in the electrical properties on the electrode were followed with a solution of 5.0 × 10^−3^ M K_3_[Fe(CN)_6_] and 5.0 × 10^−3^M K_4_[Fe(CN)_6_]) prepared in 0.10 M PBS.

### 2.2. Apparatus

Electrochemical measurements were performed using a PalmSens 4 potentiostat/galvanostat controlled by PSTrace 5.3 software. All electrochemical assays were made (at least) in triplicate (*n* = 3) (at room temperature, pH = 7.4, incubation time = 30 min). Carbon screen printed electrodes (C-SPE) were purchased from Metrohm/DropSense. All devices were connected in a switch box from PalmSens 4 to perform electrochemical assays. As a reference surface, a diffuse reflectance standard was used, and reflectance was collected at normal incidence. The size and morphology of Au NP nanoparticles were studied by SEM on FEI Quanta 400FEG ESEM/EDAX PEGASUS X4M instruments.

### 2.3. Assembly of the Glucose Sensor Material

**Figure 1:**
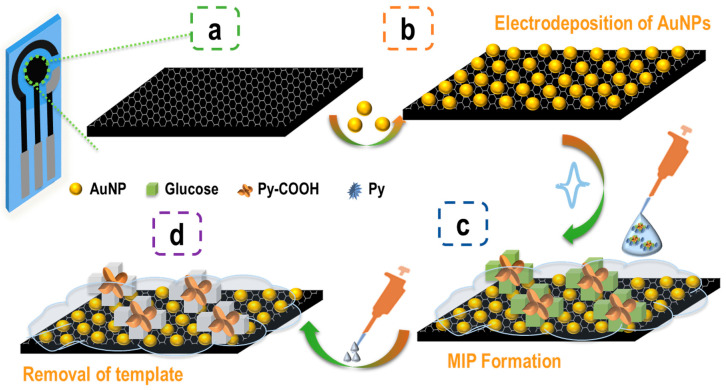
Schematic representation of the MIP sensor for glucose detection and different modifications on the working electrode. (**a**) After cleaning the surface; (**b**) electrodeposition of AuNP; (**c**) electropolymerisation of the MIP sensing layer; (**d**) removal of the glucose by incubation in water.

Figure 1 shows a schematic representation of the glucose biosensor on the carbon working electrode of the C-SPEs. The setup was carried out in several steps, each of which was followed by electrochemical measurements. First, the working electrode was cleaned by an electrochemical treatment consisting of cyclic scanning with 5-fold potential changes of −0.10 V and 1.50 V at 0.05 V/s in a 0.05M H_2_SO_4_ solution. The next step was the electrochemical preparation of the AuNPs in situ. This was done by chrono-amperometry at −1.5 V for 600 s, according to the previously identified conditions [30]. Subsequently, the MIP layer was prepared by bulk imprinting in situ with a solution prepared in PBS solution containing Glucose, Py, and Py-COOH. Electropolymerisation was performed using cyclic voltammetry (CV) in the voltage range between −0.20 V and +0.85 V at a scan rate of 0.10 V/s. The control layer containing NIP instead of MIP was prepared in a similar way, with a solution containing only Py and Py-COOH. The imprinted sites formed in the MIP became free/available for binding after the glucose was removed. This was achieved by incubating the working electrode in ultrapure water for 1 h.

### 2.4. Electrochemical Procedures

CV was performed by scanning potentials from −0.3 to +0.7 V at a scan rate of 50 mV/s. For the EIS measurements, an open-circuit sinusoidal potential perturbation with an amplitude of 0.01 V and 50 logarithmically distributed data points over a frequency range of 10–0.01 Hz was used. The EIS data were fitted to a Randles equivalent circuit using PalmSens 4 software and analysed using Nyquist plots showing the frequency response of the electrode–electrolyte system and the area plot of the imaginary component (Z″) of the impedance versus the real component (Z′). The diameter of the semicircle determined in the EIS was considered as the charge transfer resistance (Rct) [31]. Square wave voltammetry (SWV) was also used to track the changes in the MIP arrangement by scanning from −0.3 to +0.7 V at a scan rate of 20 mV/s. SWV is a fast and sensitive approach compared to the previous tests and provides large currents, which increases accuracy [32].

Electrochemical measurements of the novel glucose–MIP film were monitored by CV, EIS, and SWV measurements using a redox probe solution of 5.0 mM [Fe(CN)_6_]^3−^ and 5.0 mM [Fe(CN)_6_]^4−^ in 0.01M PBS (pH 7.4). The calibration curves were determined using EIS with standard solutions of glucose in 0.01M PBS (pH 7.4) in the range of 1.0µM to 1.0 mM. Direct glucose measurements were performed by chronoamperometry at 0.2 V/s for 100 s for each standard solution. The limit of detection (LOD) was calculated as x + 3σ, where x is the average value of the EIS blank signals (obtained in the absence of glucose) and σ is the known standard deviation of consecutive EIS blank signal measurements [33].

In selectivity studies, an MIP technology was used for glucose and various analytes that may be present in serum samples. In these experiments, two independent devices were tested. Direct electrochemical measurements were evaluated to achieve analytical performance with the same concentration range as direct detection of glucose.

### 2.5. Direct Detection for Glucose

All amperometric measurements were carried out at room temperature with constant stirring. Increasing concentrations of glucose were obtained by adding 5 to 1500 µL of an aqueous solution of 0.1 mol/L glucose to a 10 mL beaker containing 5 mL of 1.0 × 10^−2^ mol/L of a suitable buffer with a fixed pH value and a fixed ionic strength. The potentials of the stirred glucose solutions were measured at room temperature by chronoamperometry at 0.2 V for 100 s for each standard solution.

## 3. Results and Discussion

Prior to the preparation of AuNPs on the carbon electrode, the C-SPEs were pretreated by electrochemical cleaning with H_2_SO_4_. This pretreatment step had several advantages, such as reducing electrode variability, increasing the reproducibility of different electrodes from the same commercial batch, and increasing the reproducibility of the resulting biosensors. Before proceeding with each electrode, stability was confirmed by successive incubation and buffer measurements until a stable signal was achieved.

### 3.1. Production and Impact of AuNPs

Although there are several chemical methods to produce AuNPs that can control size and shape, electrochemical techniques may offer some advantages [34]. The main advantages include ease of anchoring to the surface, speed, and the fact that no chemical or binding agents are required [35]. In addition, the volume of solutions used for the electrochemical fabrication of AuNPs is very small, so little reagent is wasted. Therefore, these AuNPs are more environmentally friendly than those produced by chemical techniques. The electrochemical in situ growth also enables a more homogeneous distribution of the particles on the surface of the electrode.

AuNPs were prepared with a solution of tetrachloric acid in ultrapure water poured onto the three-electrode system (stirring and the use of nitrogen improved reproducibility) by exposing the working electrode to a constant potential for several minutes. The AuNPs formed in this way were visible to the naked eye as the surface of the working electrode glowed gold-coloured (Figure S1a). The electrochemical data confirmed this by altering the spectra of the original electrode by decreasing the Rct or increasing the current of the standard iron redox probe (Figure S1b–d). In terms of sensitivity, EIS was the most sensitive method to confirm the presence of gold. The Rct was ~seventy-seven times lower than the original value, while the current increase of the SWV was ~two times and the increase of the CV currents of the oxidation and reduction peaks was ~one time and one time, respectively.

**Figure 2:**
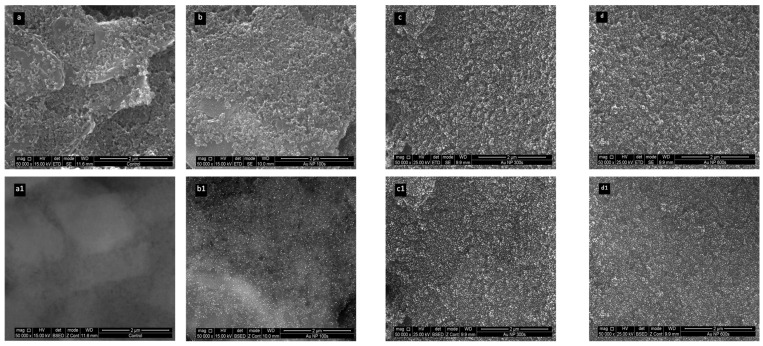
SEM images of the C-SPEs having Au NPs generated in situ by means of different electrodeposition times. (**a**) Control; (**b**) 100 s; (**c**) 300; (**d**) 600 s; and (**a1**–**d1**) SEM figures with backscattered electrons (BSEs).

SEM analyses were performed to characterise the AuNPs produced for different deposition times: 100 s (Figure 2b), 300 (Figure 2c), and 600 s (Figure 2d). These were compared with a control, which was the carbon electrode before gold plating (Figure 2a). The AuNPs were identified by the bright spots on the images (which reflected more electrons than the carbon background). These bright spots were not present in the control sample, confirming that they signalled the presence of AuNPs (Figure 2(a1–d1)). The number of AuNPs on the surface of the electrode increased with the duration of electrodeposition, as more time was available for the production of nanoparticles. The nanoparticles were homogeneously distributed on the electrode surface and exhibited small, almost hemispherical AuNPs. Overall, the best state was the one produced after 600 s of electrodeposition, as the electrical gains obtained during the electrochemical response were relevant.

Thus, the electrochemical behaviour of individual glucose solutions from −1.0 V to +1.0 V in PBS medium on electrodes with and without AuNPs was evaluated by CV assays and compared in Figure S2. Overall, it was clear that glucose was not oxidised when the scan was performed from −1.0 to +1.0 V on carbon electrodes, but an oxidative current was detected after +0.85 V when the carbon electrodes contained AuNPs, suggesting that glucose was oxidated in the presence of the AuNPs under milder conditions. In addition, the current values increased more than five-fold when the AuNPs were present.

### 3.2. Fabrication of the Sensing Layer

MIP production started with the selection of electrochemical conditions that ensured the formation of a suitable polymer layer using Py and Py-COOH. Py was the main monomer used to build the polymer network. It has excellent electrical properties and shows good conductivity [36,37]. In addition, poly (Py) has good biocompatibility, which is well suited for the fabrication of MIPs for biological molecules [38,39]. Furthermore, the literature data confirm that poly (Py) has been produced on surfaces containing noble metals such as gold [40]. When present, Py-COOH is added in a smaller amount together with glucose to form a stable complex with the aim of improving glucose binding through hydrogen bonds between glucose and the carboxyl group. It is expected that this will increase sensitivity and selectivity, as has already been observed with an approach called SPAM [41]. Bulk imprinting was used for polymerisation, where all components are mixed and polymerised on the surface of the electrode. This is a simple and effective method for a small molecule such as glucose.

In this study, electrodes with or without AuNPs prepared as previously described were used to closely observe the effects of these nanoparticles on glucose determination by MIP binding (Figure S3). Accordingly, the potential required to promote the oxidation of Py (and thus create the conditions for the formation of a polymeric network) was lower when AuNPs were present (Figure S4). However, the potential chosen for the polymerisation of Py in the presence of glucose had to ensure that the glucose was not simultaneously oxidised, as otherwise it would be irreversibly locked into the polymeric network and the imprinting would be technically impossible as the glucose would no longer be present (as it would be partially present in the form of gluconic acid). The potential range used to prepare the polymer was between −0.2 V and +0.85 V and was repeated for five cycles in the presence of AuNPs. In the absence of AuNPs, the CV was scanned from −0.2 to +0.93 V for five cycles. The effect of the presence of Py-COOH in the polymerisation process was also analysed by checking the response with and without this monomer, the polymerisation being carried out under the same conditions (as Py, with or without AuNPs). For this purpose, Py-COOH was mixed with glucose for 2 h to allow the formation of a hydrogen bond between glucose and the carboxylate group.

The final step in all strategies was to remove the template from the polymer film. For this purpose, the working electrode was incubated with ultrapure water for 1 h to ensure that the glucose had a high solubility on the electrode. In general, each modification of the surface was compared with three electrochemical CV, EIS, and SWV features in two MIPs and NIP (with and without gold nanoparticles), and the analytical performance for both strategies was analysed and the best condition was selected.

The resulting CV, EIS, and SWV data is shown in Figure S4 for C-SPEs/MIP-Py-COOH and C-SPEs/NIP-Py-COOH films without AuNPs. Overall, the CV profiles (Figure S4(a1,a2)) of the iron redox probe of the polymer film (MIP or NIP) confirm the presence of a strongly blocked surface. The redox peaks of the iron probe on the SPEs showed a stronger decrease and a larger peak-to-peak distance of the potential after the growth of the polymer film. The results of EIS (Figure S4(b1,b2)) and SWV (Figure S4(c1,c2)) are consistent with these observations.

In the comparison between MIP and NIP, the Rct increased more significantly in the MIP film. However, the other electrochemical techniques, CV (Figure S4a) and SWV (Figure S4c), showed slight differences between them, even after removing the template from the polymer layer. In general, comparison of the electrochemical CV, EIS, and SWV properties of MIPs (Figure S4(1)) and NIPs (Figure S4(2)) before and after glucose removal showed that a significant change was observed in the NIP films before and after exposure to ultrapure water and that the MIP material exhibited more modification, especially in EIS, which decreased the Rct value. Overall, these results confirmed the ineffective removal of glucose from the MIP films and the instability of the polypyrrole film when exposed to ultrapure water incubation. After assembling the MIP and NIP devices, the analytical performance was tested by incubating several glucose standard solutions with increasing concentrations on the working electrode by tracking the electrical EIS properties of a standard iron redox probe after each incubation. For this purpose, a drop of a glucose standard solution was left for a 30-min incubation time. Figure S5 shows the random behaviour of MIP (Figure S5a) and NIP (Figure S5b) when the glucose concentration is increased. Figure S5c,d correspond to the calibration curves and also show the random and non-random response to the sensors of Rct versus the logarithm (glucose concentration).

**Figure 3:**
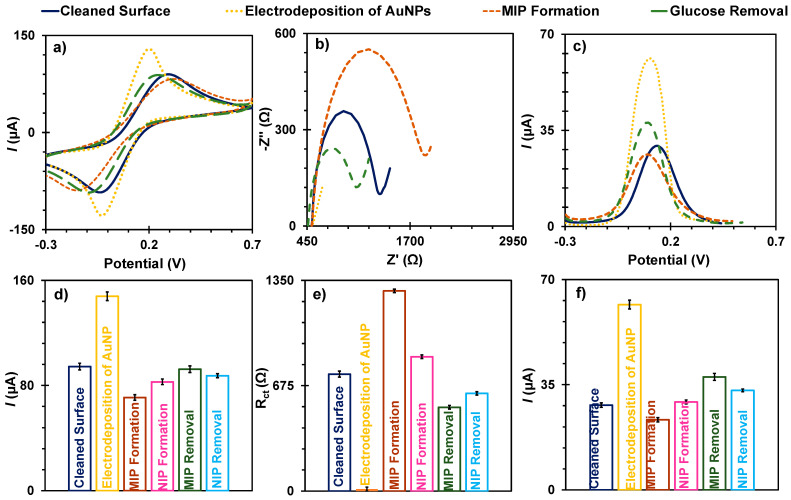
Electrochemical data upon the biosensor construction, using CV (**a**), EIS (**b**), and SWV (**c**) measurements. Data include after cleaning the surface, after electrodeposition of AuNPs, electropolymerisation of MIP film (with Py and Py-COOH), and removal of the template. Graphics (**d**,**f**) show the current peak’s intensity, and graphic (**e**) presents the R_ct_ values with the respective error bars, highlighting the reproducibility of three independent devices.

In the second method, the gold nanoparticles were electrodeposited on the surface, and each modification was monitored with CV, EIS, and SWV (Figure 3). After pre-cleaning the surface, the gold nanoparticles were electrodeposited in situ. The data showed a higher average CV peak current (Figure 3a), which was achieved in the previous cleaning phase. The cathodic peak current after cleaning was ~94.38 µA lower than after electrodeposition of the AuNP and was ~147.83 µA (Figure 3d). The Rct in the EIS (Figure 3b) became negligible, confirming the improved electrical properties of the sensor surface of ~12.98 Ω. The SWV data agreed with the previous observations and showed a current increase of ~61.69 µA. The next step was to prepare the MIP film by electropolymerisation of pyrrole using CV at –0.2 V to +0.85 V for five cycles in the presence of template glucose. The negative control (NIP) was prepared in a similar way, but in the absence of glucose.

Overall, the presence of a polymer film showed a decrease in current in the CV (Figure 3a) and SWV (Figure 3c) measurements, as poly (pyrrole) signalled that the surface was blocked. Although pyrrole is a polymer with a high conductivity, this property depends on the pH effect and the electrolyte [42]. The results of the EIS spectra (Figure 3b) are consistent with these observations.

The current drop is more pronounced in the MIP, indicating the presence of glucose (Figure 3a), which hinders the occurrence of the redox reaction causing this current drop. The current at the cathodic tip of the MIP device was lower at 71.6 µA than that of NIP at 82.73 µA (Figure 3d), although both materials have a similar CV profile due to the small size of the molecules. The Rct value in the EIS technique (Figure 3b) increased more significantly in the MIP layer at ~1283 Ω than in the NIP layer at ~860.3 Ω (Figure 3e) and the SWV measurement (Figure 3f) showed a more significant decrease in the MIP layer at ~23.31 µA than in the NIP layer at ~29.23 µA.

After the polymerisation phase, the electrodes were incubated in ultrapure water for 1 h. Ultrapure water was chosen because glucose is very soluble in water and can therefore be extracted from the polymer network by simple dissolution. The choice of water also ensured the integrity of the polymer. As for the CV profile, the NIP films showed a small increase in current at ~87.4 µA compared to MIP at ~92.34 µA (Figure 3d). In the EIS spectra, NIP showed a decrease at ~625.1 Ω compared to the MIP device, which showed the highest decrease at ~536.1 Ω (Figure 3e), and the SWV data is consistent with the other measurements (Figure 3f), with MIP showing a highest increase at ~37 µA compared to NIP ~33 µA. Overall, these results confirm the effective removal of glucose from the MIP films and the stability of the poly (Py) film when exposed to high purity water using AuNP, increasing the surface area and enhancing sensitivity.

### 3.3. Electrochemical Analytical Performance

**Figure 4:**
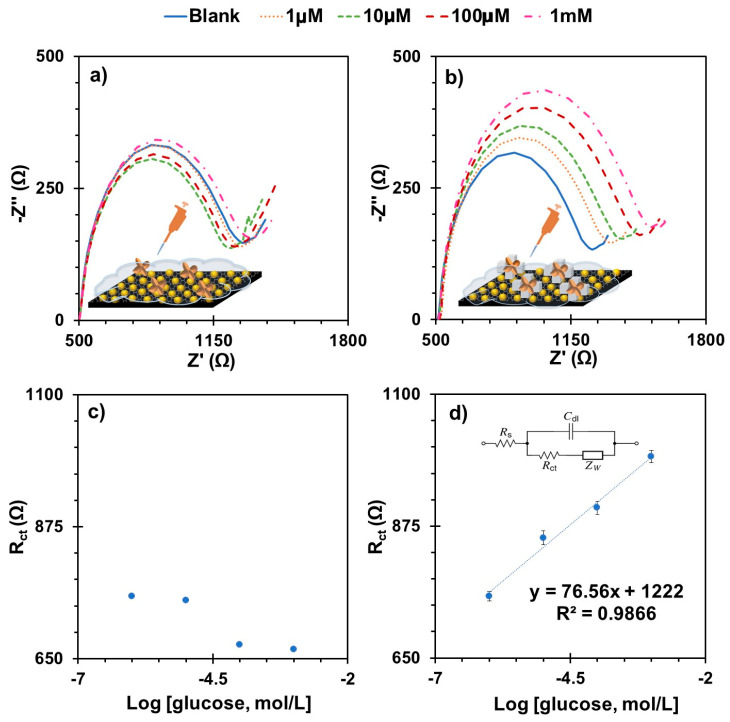
Calibration curves of the biosensors C-SPEs/AuNPs/MIP-NIP-Py-COOH, (**a**) with EIS measurements of NIP devices and (**c**) the corresponding calibration curves; (**b**) EIS measurements of MIP devices and the corresponding calibration curves (**d**), obtained in 5.0 mM [Fe(CN)_6_]^3−^ and 5.0 mM [Fe(CN)_6_]^4−^ solution prepared in PBS buffer.

The analytical performance of the device was evaluated by incubating a standard glucose solution on the MIP for 30 min, then washing it out and replacing it with the standard iron redox probe to extract the corresponding Nyquist plot (Figure 4b). This procedure was repeated successively for increasing concentrations of glucose after the required number of times with buffer until the signal was stable.

The linear response was observed from 1.0 μM to 1.0 mM (0.02 to 18.02 mg/dL) by plotting Rct against the logarithm. The average slope of three independent readings was 73.9 Ω/decade (Figure 4d), with a maximum standard deviation of 3.4% and an LOD of 0.15 µM (3 μg/dL). The calibration curves showed squared correlation coefficients > 0.99, confirming the excellent quality of the linear response. The reproducibility was also excellent, considering that the RSD values were between 1.37% (minimum) and 3.2% (maximum) within the linear response range.

The same calibration approach was used for the NIP to understand the ability of the imprinted sites on the electrode surface to bind glucose (Figure 4a). The random response of the NIP to increasing glucose concentrations confirmed that the electrochemical response of the MIP was dominated by the imprinted binding sites and that non-specific binding to the poly (Py) was negligible (Figure 4c).

### 3.4. Direct Electrochemical Readings of Glucose

The direct measurement of glucose was tested using chronoamperometry to avoid the use of a redox probe when performing quantitative measurements with the C-SPs/AuNPs/MIP devices. Chronoamperometry was performed at +0.2 V as the AuNPs were present on the surface. They catalysed the oxidation of glucose and thereby reduced the potential required for this oxidation (without AuNPs, no current signals would be generated at this potential, as shown in Figure S1).

**Figure 5:**
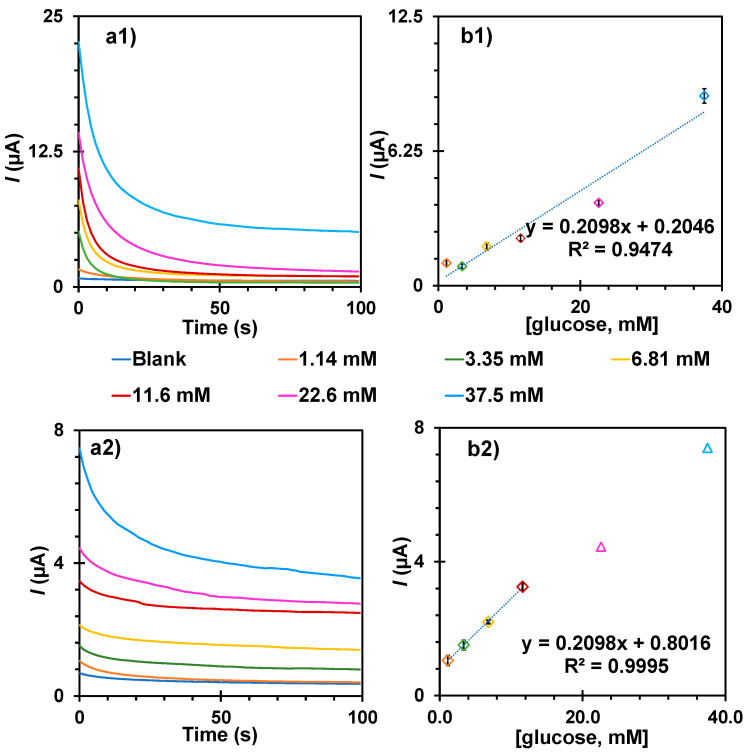
Chronoamperometric data (**a**) of direct readings of glucose (**1**) on the C-SPEs/MIP-Py-COOH film and (**2**) on the C-SPEs/AuNPs/MIP-Py-COOH film, along with the corresponding representative calibration curves in t = 20 s (**b**).

The typical data we obtained are shown in Figure 5 for the specific time t = 20 s. The calibration curves show a higher sensitivity when using an MIP technology at different time points of the chronoamperometry in Figure S6.

The importance of the presence of MIP for the electrochemical readings was confirmed by testing the response of glucose directly on a substrate without MIP (C-SPs/AuNPs). As can be seen in Figure 5a, the maximum current intensity was approximately three times greater than in a device containing the MIP, as the conductivity of the sensor area was blocked by the polymer. However, it should be kept in mind that selective oxidation of glucose under the chosen potential is not possible, as other compounds that can oxidise at the same potential would also generate current signals.

**Figure 6:**
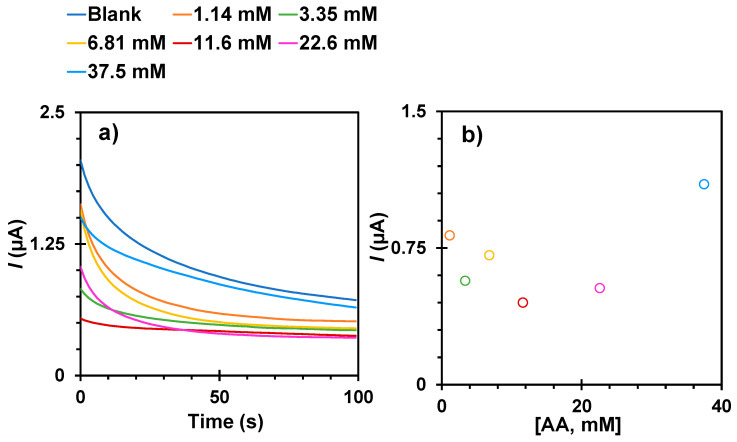
Chronoamperometric data (**a**) of direct readings of AA on the C-SPEs/AuNPs/MIP-Py-COOH film along with the (**b**) corresponding representative calibration curves in t = 20 s.

Therefore, the ability of the MIP to selectively discriminate glucose from other compounds was tested by evaluating the response of the C-SPs/AuNPs/MIP against AA standard solutions, as AA is a known interfering compound in glucose measurements. In this study, AA solutions were prepared at the same concentrations as measured for glucose, i.e., between 1.14 and 37.5 mM. The results obtained are shown in Figure 6 and show a randomised response. The maximum initial current levels were also approximately four times lower than for a direct glucose measurement, implying that AA is excluded from the polymeric network and moves too far away from the AuNPs to be efficiently oxidised. It is important to note that the AA content in serum is normally between 0.4 and 1.7 mg/dL (22 to 85 µM), which is much lower than the concentration tested, meaning that the interference from AA in a true serum analysis is negligible.

Overall, the analytical data generated by chronoamperometry show a linear trend of current versus concentration, from 1 to ~40 mM for a direct glucose measurement. This clearly shows that the use of a redox probe increases the capacity of the same electrode in terms of detection capability and sensitivity. By using EIS and a redox probe, the C-SPs/AuNPs/MIP is able to achieve ~1000× less glucose over a wider concentration range than a linear response in the 1 to 1000 μM. Considering that the sample is always diluted to ensure that the sample contains a similar electrolyte content in terms of pH and ionic strength, the use of a redox probe seems more appropriate. The normal fasting blood glucose level is between 70 mg/dL (3.9 mM) and 100 mg/dL (5.6 mM), which means that the sample dilution allowed with this measurement method is much higher.

### 3.5. Analytical Performance in Human Sera

The C-SPs/AuNPs/MIP electrode was evaluated in glucose standard solutions prepared in buffer and diluted human serum (1:1000) to assess the ability of the system to accurately analyse real samples. All assays were analysed in triplicate, and a typical Nyquist plot is shown in Figure S7. Given the sensitivity of the signals obtained, it is obvious that the slope is greater when dilute human serum solutions were used compared to buffered solutions, but the relative data show otherwise. Calibrations plotting the ‘standard solution Rct/blank Rct’ ratio show a similar trend for buffered standard solutions and buffered standard solutions with diluted serum, indicating that the analytical sensitivity was similar (Figure S8). The limit of detection (LOD) calculated from the Nyquist plots was 0.11 µM, and the linear trend indicates that the biosensor can be used to analyse human sera from 1000 µM to 1000 mM. For control purposes, the response of the NIP to these diluted serum solutions was also investigated. A random response was observed, confirming the critical role of the MIP on the working electrode.

These results confirm that the MIP layer maintains sensitive detection of glucose even in the presence of biological components. Compared to other reported sensors, the analytical performance of the present system is highly competitive. For example, Kim et al. (2017) [21] developed an MIP-based glucose sensor modified with gold nanoparticles and a functional terthiophene polymer, which achieved a detection limit of 0.19 µM. Sehit et al. (2020) [28] presented a highly sensitive MIP-AuNP-based sensor with an ultralow LOD of 1.25 nM. Fan et al. (2018) [29] reported an optical AuNP-based sensor with a linear range of 2.4 to 30.2 mM. The sensor developed in this study offers a favourable compromise between simplicity, sensitivity, and accuracy, with an extensive linear range of response (1.0 µM to 1.0 mM) and a low detection limit of 0.11 µM.

Overall, in situ electropolymerisation of the MIP layer and electrochemical generation of gold nanoparticles streamline the sensitivity while keeping material and operating costs low. These advantages make the proposed sensor a promising candidate for enzyme-free glucose analysis in real biological fluids.

## 4. Conclusions

This work demonstrates the successful development of a simple, innovative, highly sensitive and selective MIP material for the detection of glucose on a carbon substrate modified with AuNPs. This simple design includes all steps assembled in situ with great advantages in terms of the catalytic effect of AuNPs in the oxidation of glucose. This was confirmed and optimised by selecting the best conditions for the in situ generation of the nanoparticles, improving the sensitivity and using MIP technology to improve the selectivity of the sensor. The effect of the electrodeposited nanomaterial on the performance of the sensor was demonstrated by the parallel study of the response without this metal and confirmed that glucose could not be detected in a linear trend without the NPs. The C-SPE/AuNP/MIP/Py-COOH sensor showed high selectivity towards glucose.

Overall, this electrochemical biosensor is easy to construct and can be used for dual detection, which includes both optical and electrochemical measurements and should provide good sensitivity.

## Figures and Tables

**Table 1:** List of glucose sensors using MIP-based sensing systems, along with their main analytical features.

Electrochemical Method	Functional Monomer/Cross-Link	Nanomaterial/Substrate	Linear Range	LOD	Surface Characterisation	Cross-Reactivity Testing	References
CV	VPBA, MBA	ITO electrode	Up to 900 mg/dL	-	XPS	-	Yoshimi et al., 2009 [23]
CV	Silicon iil on a copper wire	PVA electrodes by MnO_2_/CuO on GO NPs	0.5 to 4.4 mM	53 µM	XRD; FTIR; SEM; EDX	-	Farid et al., 2016 [24]
CV; EIS	MAA, MBA, 4-VP	Porous Ni foam	10 to 55 mM	-	Raman; SEM	DA, FRU, Glucose, AA, AP	Li et al., 2017 [25]
LSV; ChronAMP	Nafion, APBA, Polyurethane	CuCo bimetal-coated on SPCE	1.0 µM to 25.0 mM	0.65 µM	FE-SEM; EDXS; XRD	MAN, LAC, UA, AP, DA, AA, Cysteine, GAL, FRU; XYL; SUC, LAA; MAL	Cho et al., 2018 [15]
ChronAMP	VPBA, PBA	FET	100 μM to 4 mM	3.0 µM	FTIR; XPS	FRU; SUC	Kajisa et al., 2018 [26]
CV, DPV, EIS	AAM, NNMBA	Au-SPE	0.5 to 50 μg/mL	0.59 μg/mL	AFM; SEM-EDS	LAC, SUC	Diouf et al., 2019 [22]
CV, EIS	AAM	Porous Ni foam	0.8 to 4.0 mM	0.45 mM	SEM; XRD	AA; FRU; AP	Wu et al., 2019 [27]
EIS	AAM, EGDMA		14.4–330 μM				
CV, EIS, SWV, UV-Vis	o-PD, Scopoletin	AuNPs	1.25 nM to 2.56 μM	1.25 nM	AFM; SEM; QCM; TEM	SUC, DA; starch, BSA	Sehit et al., 2020 [28]
CV, EIS	pTBA	AuNPs/SPCE	0.32 µM to 1.0 mM	0.19 µM	AFM; XPS; QCM	GAL; DA; MAL; FRU, LAC, MAN, XYL; RIB, AP; UA; SUC; AA	Kim et al., 2017 [21]
UV-Vis	APBA	AuNP	2.4 to 30.2 mM	-	TEM; SEM	CHOL, UA	Fan et al., 2018 [29]

4-VP: 4-Vinyl Pyridine; AA: Ascorbic Acid; AAM/NNMBA: Acrylamide/Bis-Acrylamide; AFM: Atomic Force Microscope; AP: Acetamidophenol; APBA: Aminophenyl Boronic Acid; AuNPs: Gold Nanoparticles; Au-SPE: Gold-Screen Printed Electrode; BSA: Bovine Serum Albumin; CHOL: Cholesterol; ChronAMP: Chronoamperometry; CV: Cyclic Voltammetry; DA: Dopamine; DPSV: Differential Pulse Stripping Voltammetry; DPV: Differential Pulse Voltammetry; EDX: Energy-Dispersive X-Ray Spectroscopy; EDXS: Energy-Dispersive X-Ray Spectroscopy; EGDMA: Ethylene Glycol Dimethacrylate; EIS: Electrochemical Impedance Spectroscopy; FE-SEM: Field Emission Scanning Electron Microscope; FET: Field-Effect Transistor; FRU: Fructose; FTIR: Fourier-Transform Infrared Spectroscopy; GAL: Galactose; GLU: Glucose; GO: Graphene Oxide; GQD: Graphene Quantum Dots; Gr: Graphene; ITO: Indium Tin Oxide; LAC: Lactose; LSV: Linear Sweep Voltammetry; MAA: Methacrylic Acid; MAL: Maltose; MAN: Mannose; MBA: Methylene Bisacrylamide; MBA: N,N’-Methylene Bisacrylamide; NPs: Nanoparticles; *o*-PD: Ortho-Phenylenediamine; PBA: Phenylboronic Acid; PSS: Poly (styrene sulfonate); pTBA: Benzoic Acid-Functionalised Poly (terthiophene); PVA: Polyvinyl Acetate; Py: Pyrrol; QCM: Quartz Crystal Microbalance; RIB: Ribose; SEM: Scanning Electron Microscopy; SPCE: Screen Printed Carbon Electrode; SUC: Sucrose; SWV: Square Wave Voltammetry; TEM: Transmission Electron Microscopy; TGA: Thermogravimetric Analysis; TMB: Tetramethylbenzidine; UA: Uric Acid; VPBA: 4-vinylphenylboronic acid; XPS: X-Ray Photoelectron Spectroscopy; XRD: X-Ray Diffraction; XYL: Xylose.

## Data Availability

The original contributions presented in this study are included in the article/Supplementary Materials. Further inquiries can be directed to the corresponding author.

## Supplementary Materials

The following supporting information can be downloaded at: https://www.mdpi.com/article/10.3390/bios15080537/s1, Figure S1: Electrochemical data after cleaning and after electrodeposition of AuNPs (a), using CV (b), EIS (c) and SWV (d). Figure S2: Voltammograms of Py and glucose solutions prepared in PBS with (a) and with (b) without AuNPs. Figure S3: Schematic representation the several optimizations. Figure S4: Electrochemical measurements of CV (a), EIS (b) and SWV (c) in 5.0 × 10^−3^ M [Fe(CN)_6_ ]^3−^ and 5.0 × 10^−3^ M [Fe(CN)_6_]^4−^ solution, prepared in PBS, of the NIP (1) and MIP (2) films (without AuNPs/MIP-Py-COOH), at the several steps of the biosensor assembly. Figure S5: Nyquist plots of (a) MIP and (b) in NIP sensors (C-SPEs/without AuNPs/MIP-Py-COOH) in 5.0 × 10^−3^ M [Fe(CN)_6_]^3−^ and 5.0 × 10^−3^ M [Fe(CN)_6_]^4−^, after incubation in standard solutions of glucose of increasing concentrations, prepared in PBS buffer, and the corresponding calibration curves (c and d). Figure S6: Chronoamperometric data (a) of direct readings of glucose on the C-SPEs/MIP-Py-COOH film with the corresponding representative calibration curves (b) in different time points: 0 s (b1); 20 s (b2) and 100 s (b3). Figure S7: Calibration curves of the biosensors C-SPEs/AuNPs/MIP-NIP-Py-COOH, with (a) EIS measurements of NIP devices and (c) the corresponding calibration curves; (b) EIS measurements of MIP devices and the corresponding calibration curves (d) in serum 1000× diluted. Readings obtained in 5.0 × 10^−3^ M [Fe(CN)_6_]^3−^ and 5.0 × 10^−3^ M [Fe(CN)_6_]^4−^ solution prepared in PBS buffer, after incubation in increasing concentrations of glucose standard solutions. Figure S8: Calibration curves of the biosensors C-SPEs/AuNPs/MIP-NIP-Py-COOH, with EIS measurements in buffer (a) and serum (b) and the corresponding calibration curves with relative values (c) in buffer and (d) serum 1000× diluted. Readings obtained in 5.0 × 10^−3^ M [Fe(CN)_6_]^3−^ and 5.0 × 10^−3^ M [Fe(CN)_6_]^4−^ solution prepared in PBS buffer, after incubation in increasing concentrations of glucose standard solutions in different medium (buffer and serum).

## References

[1] Fauziah S., Karlsson A., Gustavsson H. (2017). Density Functional Theory (DFT) Study of Molecularly Imprinted Polymer (MIP) Methacrylic Acid (MAA) with D-Glucose. IOP Conf. Ser. Mater. Sci. Eng..

[2] Cai T., Gao Y., Yan J., Wu Y., Di J. (2017). RSC Advances nanoplates and gold nanoparticles. RSC Adv..

[3] Wang H., Lee A. (2015). ScienceDirect Recent developments in blood glucose sensors. J. Food Drug Anal..

[4] Bruen D., Delaney C., Florea L. (2017). Diamond, Glucose Sensing for Diabetes Monitoring: Recent Developments. Sensors.

[5] Gusev M., Poposka L., Spasevski G., Kostoska M., Koteska B., Simjanoska M., Ackovska N., Stojmenski A., Tasic J., Trontelj J. (2020). Review Article Noninvasive Glucose Measurement Using Machine Learning and Neural Network Methods and Correlation with Heart Rate Variability. J. Sens..

[6] Sabu C., Henna T.K., Raphey V.R., Nivitha K.P., Pramod K. (2019). Biosensors and Bioelectronics Advanced biosensors for glucose and insulin. Biosens. Bioelectron..

[7] Teymourian H., Barfidokht A., Wang J. (2020). Electrochemical glucose sensors in diabetes management: An updated review (2010–2020). Chem. Soc. Rev..

[8] Clark L.C., Lyons C. (1962). Electrode Systems for Continuous Monitoring in Cardiovascular Surgery. Ann. N. Y. Acad. Sci..

[9] Gonzales W.V., Mobashsher A.T., Abbosh A. (2019). The Progress of Glucose Monitoring—A Review of Invasive to Minimally and Non-Invasive Techniques, Devices and Sensors. Sensors.

[10] Peng Z., Xie X., Tan Q., Kang H., Cui J., Zhang X., Li W., Feng G. (2022). Blood glucose sensors and recent advances: A review. J. Innov. Opt. Heal. Sci..

[11] Free A.H., Free H.M. (1984). Self testing, an emerging component of clinical chemistry. Clin. Chem..

[12] Henning T., Cunningham D.D., Stenken J.A. (2009). Commercially Available Continuous Glucose Monitoring Systems. In Vivo Glucose Sensing.

[13] Asghar N., Mustafa G., Yasinzai M., Al-soud Y.A. (2019). Real-Time and Online Monitoring of Glucose Contents by Using Molecular Imprinted Polymer-Based IDEs Sensor. Appl. Biochem. Biotechnol..

[14] Nguyen H.H., Lee S.H., Lee U.J., Fermin C.D., Kim M. (2019). Immobilized Enzymes in Biosensor Applications. Materials.

[15] Je S., Noh H., Won M., Cho C., Bok K. (2018). A selective glucose sensor based on direct oxidation on a bimetal catalyst with a molecular imprinted polymer. Biosens. Bioelectron..

[16] van Enter B.J., Von Hauff E. (2018). Challenges and perspectives in continuous. Chem. Commun..

[17] Belbruno J.J. (2018). Molecularly Imprinted Polymers. Chem. Rev..

[18] Razak K.A., Zulkifli Z.A., Ridhuan N.S., Nor N.M. (2017). The effect of gold nanoparticles modified electrode on the glucose sensing performance. AIP Conf. Proc..

[19] Caldara M., Kulpa J., Lowdon J., Cleij T., Diliën H., Eersels K., van Grinsven B. (2023). Recent Advances in Molecularly Imprinted Polymers for Glucose Monitoring: From Fundamental Research to Commercial Application. Chemosensors.

[20] Vaidya A., Sahoo J., Shende P. (2025). Molecularly imprinted polymer-based biosensor for detection of salivary glucose in diabetes. Int. J. Pharm..

[21] Kim D., Moon J., Lee W., Yoon J., Soo C. (2017). A potentiometric non-enzymatic glucose sensor using a molecularly imprinted layer bonded on a conducting polymer. Biosens. Bioelectron..

[22] Diouf A., Bouchikhi B., El N. (2019). A nonenzymatic electrochemical glucose sensor based on molecularly imprinted polymer and its application in measuring saliva glucose. Mater. Sci. Eng. C.

[23] Yoshimi Y., Narimatsu A., Nakayama K., Sekine S., Hattori K., Sakai K. (2009). Development of an enzyme-free glucose sensor using the gate effect of a molecularly imprinted polymer. J. Artif. Organs.

[24] Farid M., Goudini L., Piri F., Zamani A., Saadati F. (2016). Molecular imprinting method for fabricating novel glucose sensor: Polyvinyl acetate electrode reinforced by MnO_2_/CuO loaded on graphene oxide nanoparticles. Food Chem..

[25] Li X., Niu X.H., Wu H.Y., Meng S.C., Zhang W.C., Pan J.M., Qiu F.X. (2017). Impedimetric Enzyme-Free Detection of Glucose via a Computation-Designed Molecularly Imprinted Electrochemical Sensor Fabricated on Porous Ni Foam. Electroanalysis.

[26] Kajisa T., Sakata T. (2018). Molecularly Imprinted Artificial Biointerface for an Enzyme-Free Glucose Transistor. ACS Appl. Mater. Interfaces.

[27] Wu H., Tian Q., Zheng W., Jiang Y., Xu J., Li X., Zhang W., Qiu F. (2019). Non-enzymatic glucose sensor based on molecularly imprinted polymer: A theoretical, strategy fabrication and application. J. Solid State Electrochem..

[28] Sehit E., Drzazgowska J., Buchenau D., Yesildag C., Lensen M., Altintas Z. (2020). Ultrasensitive nonenzymatic electrochemical glucose sensor based on gold nanoparticles and molecularly imprinted polymers. Biosens. Bioelectron..

[29] Fan L., Lou D., Wu H., Zhang X., Zhu Y., Gu N., Zhang Y. (2018). A Novel AuNP-Based Glucose Oxidase Mimic with Enhanced Activity and Selectivity Constructed by Molecular Imprinting and O_2_-Containing Nanoemulsion Embedding. Adv. Mater. Interfaces.

[30] Serrano V.M., Silva I.S.P., Cardoso A.R., Sales M.G.F. (2022). Carbon Electrodes with Gold Nanoparticles for the Electrochemical Detection of miRNA 21-5p. Chemosensors.

[31] Chang B., Park S. (2010). Electrochemical Impedance Spectroscopy. Annu. Rev. Anal. Chem..

[32] Dauphin-Ducharme P., Arroyo-Curras N., Kurnik M., Ortega G., Li H., Plaxco K.W. (2017). A Simulation-based Approach to Determining Electron Transfer Rates using Square-Wave Voltammetry. Langmuir.

[33] Harvey D., Kane K. (2000). Modern Analytic Chemistry. Modern Analytical Chemistry.

[34] Tonelli D., Scavetta E., Gualandi I. (2019). Electrochemical Deposition of Nanomaterials for Electrochemical Sensing. Sensors.

[35] Chiang H.-C., Wang Y., Zhang Q., Levon K. (2019). Optimization of the Electrodeposition of Gold Nanoparticles for the Application of Highly Sensitive. Biosensors.

[36] Kan X., Xing Z., Zhu A., Zhao Z., Xu G., Li C., Zhou H. (2012). Molecularly imprinted polymers based electrochemical sensor for bovine hemoglobin recognition. Sens. Actuators B Chem..

[37] Cardoso A.R., Tavares A.P.M., Sales M.G.F. (2018). In-situ generated molecularly imprinted material for chloramphenicol electrochemical sensing in waters down to the nanomolar level. Sens. Actuators B Chem..

[38] Gomes R.S., Gomez-Rodríguez B.A., Fernandes R., Sales M.G.F., Moreira F.T.C., Dutra R.F. (2021). Plastic antibody of polypyrrole/multiwall carbon nanotubes on screen-printed electrodes for cystatin C detection. Biosensors.

[39] Rebelo T.S.C.R., Costa R., Brandão A.T.S.C., Silva F., Sales M.G.F., Pereira C.M. (2019). Molecularly imprinted polymer SPE sensor for analysis of CA-125 on serum. Anal. Chim. Acta.

[40] Ramanavičius A., Ramanavičiene A., Malinauskas A. (2006). Electrochemical sensors based on conducting polymer-polypyrrole. Electrochim. Acta.

[41] Moreira F.T.C., Sharma S., Dutra R.A.F., Noronha J.P.C., Cass A.E.G., Sales M.G.F. (2013). Smart plastic antibody material (SPAM) tailored on disposable screen printed electrodes for protein recognition: Application to myoglobin detection. Biosens. Bioelectron..

[42] Sadki S., Schottland P., Brodie N., Sabouraud G. (2000). The mechanisms of pyrrole electropolymerization. Chem. Soc. Rev..

