# Supplementary material for: Enzyme-Free Monitoring of Glucose Using Molecularly Imprinted Polymers and Gold Nanoparticles
Source: Biosensors (Basel). 2025 Aug 15;15(8):537. doi: 10.3390/bios15080537 (PMC12384395; doi:10.3390/bios15080537)
Supplement: Supplementary file 1 [file biosensors-15-00537-s001.zip › biosensors-3721842-supplementary.pdf]

Supplementary information

to

Enzyme-Free Monitoring of Glucose Using Molecularly Imprinted Polymers and Gold Nanoparticles

Ana Rita Aires Cardoso [1,2], Pedro Miguel Cândido Barquinha [2] and Maria Goreti Ferreira Sales [1,*]

[1] BioMark@UC/CEMMPRE (Centre for Mechanical Engineering, Materials and Processes)-ARISE (Advanced Production and Intelligent Systems), Faculty of Sciences and Technology, Department of Chemical
Engineering, University of Coimbra, 3030-790 Coimbra, Portugal; uc45752@uc.pt

[2] CENIMAT|i3N, Department of Materials Science, School of Science and Technology, NOVA University Lisbon and CEMOP/UNINOVA, Campus de Caparica, 2829-516 Caparica, Portugal; pmcb@fct.unl.pt

* Correspondence: goreti.sales@gmail.com or maria.sales@uc.pt; Tel.: +351-239-798-733

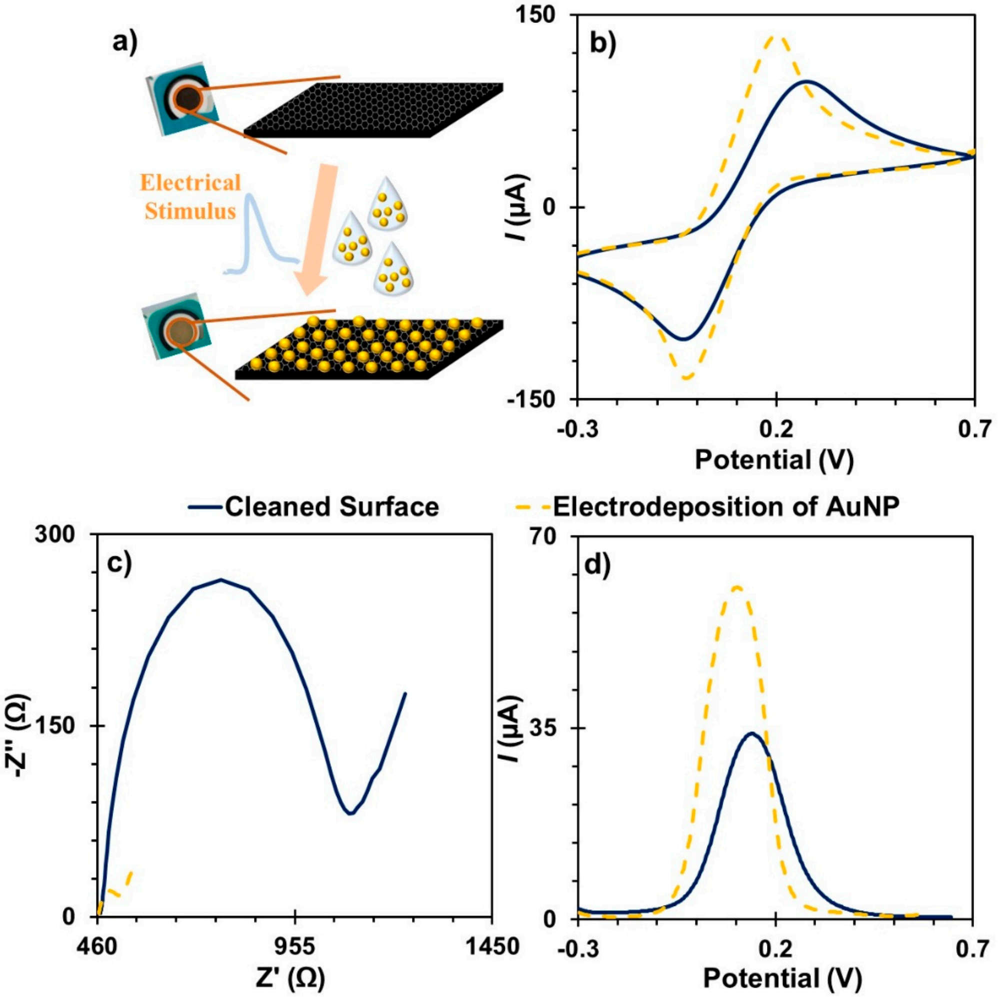

Figure S1 : Electrochemical data after cleaning and after electrodeposition of AuNPs (a), using CV(b), EIS (c) and SWV (d).

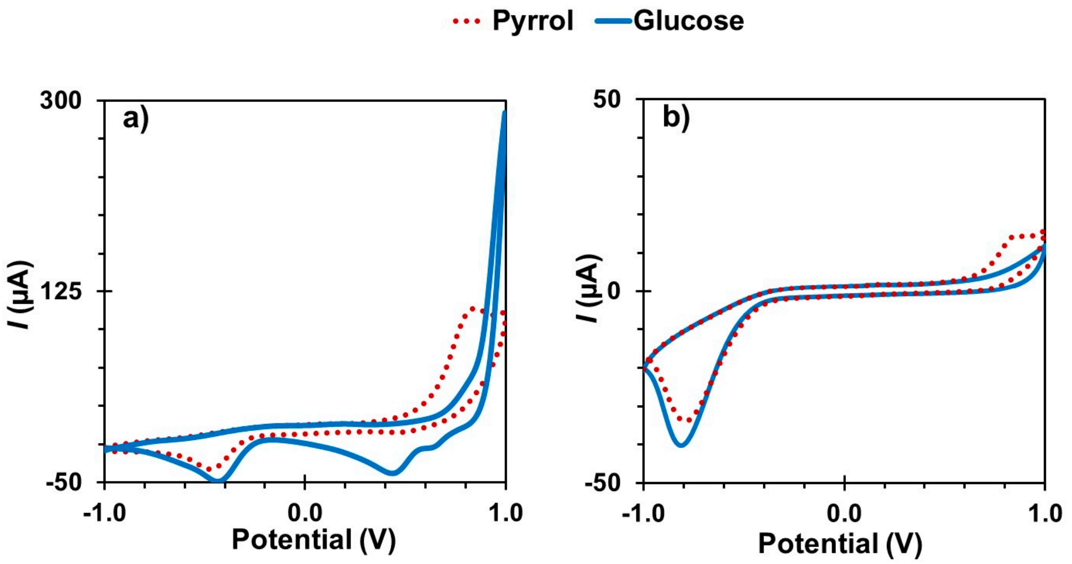

Figure S2: Voltammograms of Py and glucose solutions prepared in PBS with (a) and with (b) without AuNPs.

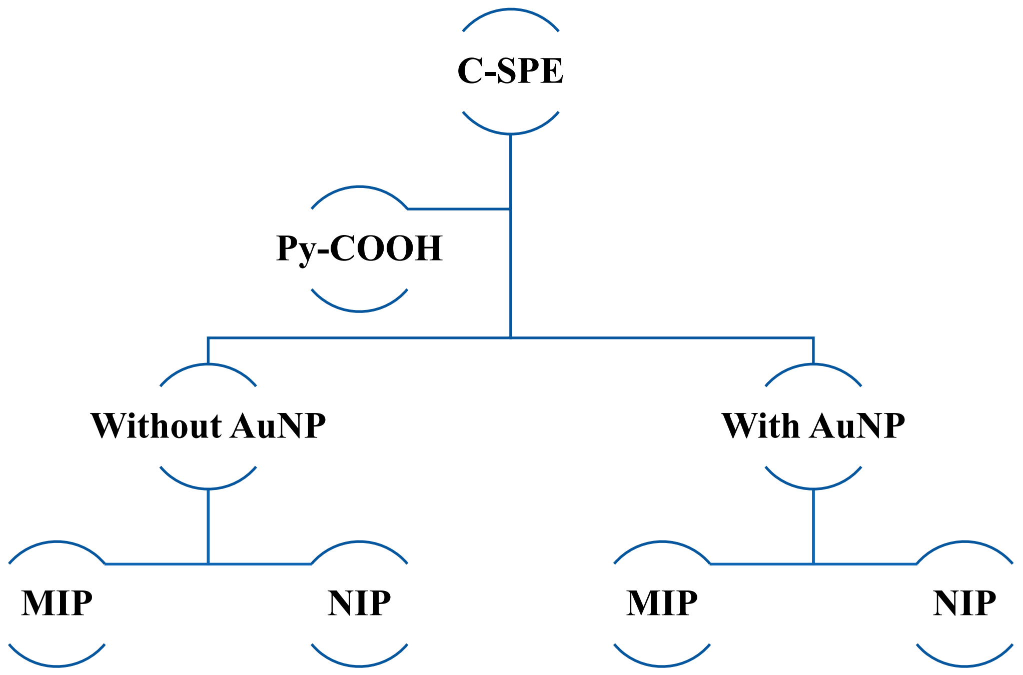

Figure S3: Schematic representation the several optimizations.

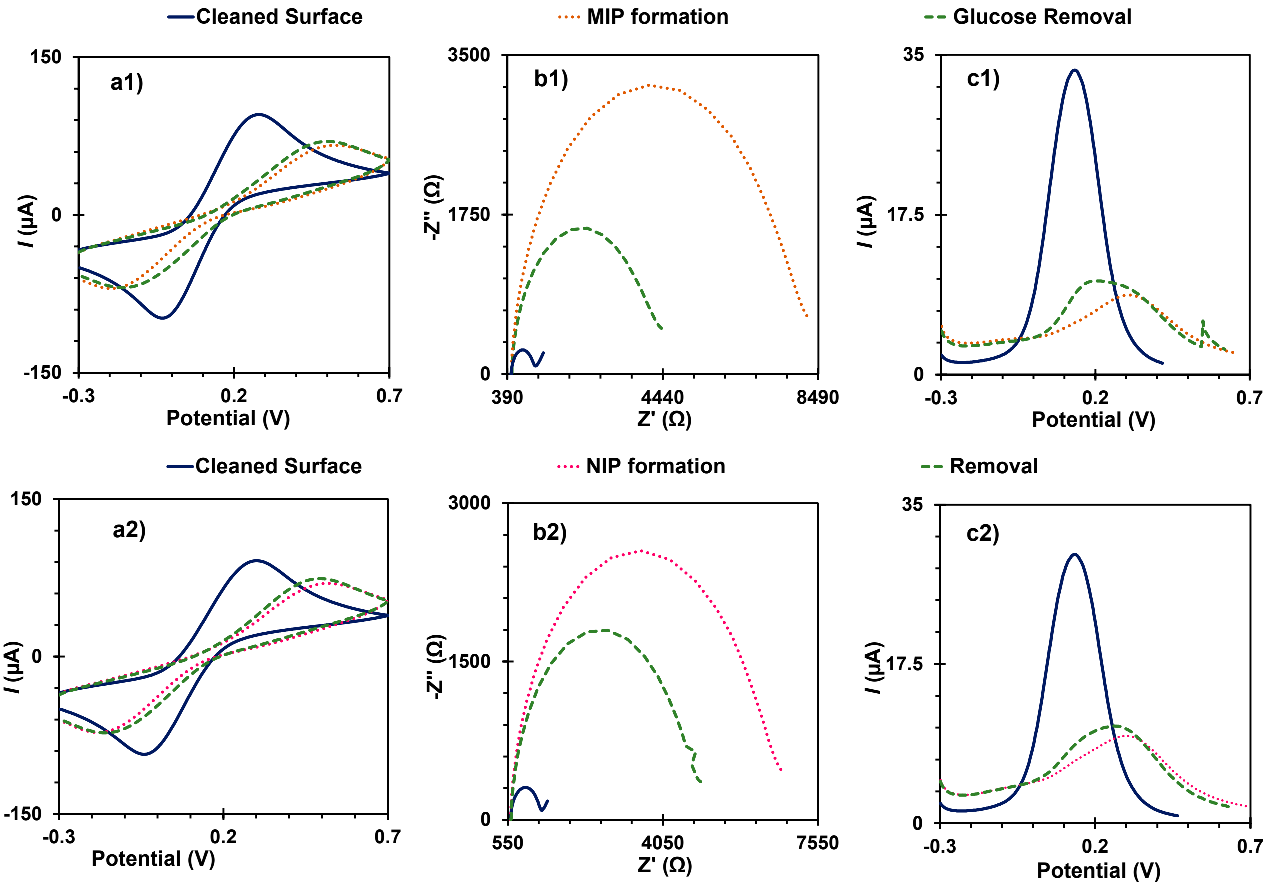

Figure S4: Electrochemical measurements of CV (a), EIS (b) and SWV (c) in 5.0×10^{-3} M $[Fe(CN)_6]^{3-}$ and 5.0×10^{-3} M $[Fe(CN)_6]^{4-}$ solution, prepared in PBS, of the NIP (1) and MIP (2) films (without AuNPs/MIP-Py-COOH), at the several steps of the biosensor assembly.

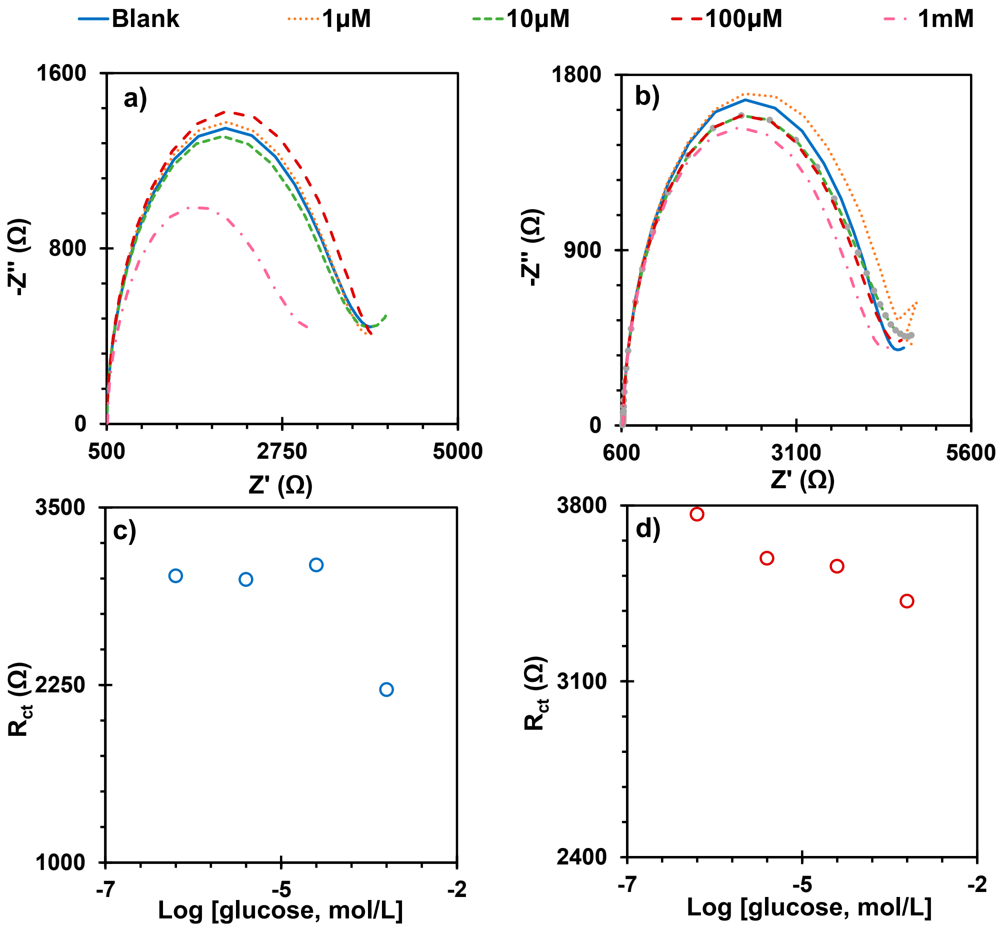

Figure S5: Nyquist plots of (a) MIP and (b) in NIP sensors (C-SPEs/without AuNPs/MIP-Py-COOH) in 5.0×10^{-3} M $[Fe(CN)_6]^{3-}$ and 5.0×10^{-3} M $[Fe(CN)_6]^{4-}$, after incubation in standard solutions of glucose of increasing concentrations, prepared in PBS buffer, and the corresponding calibration curves (c and d).

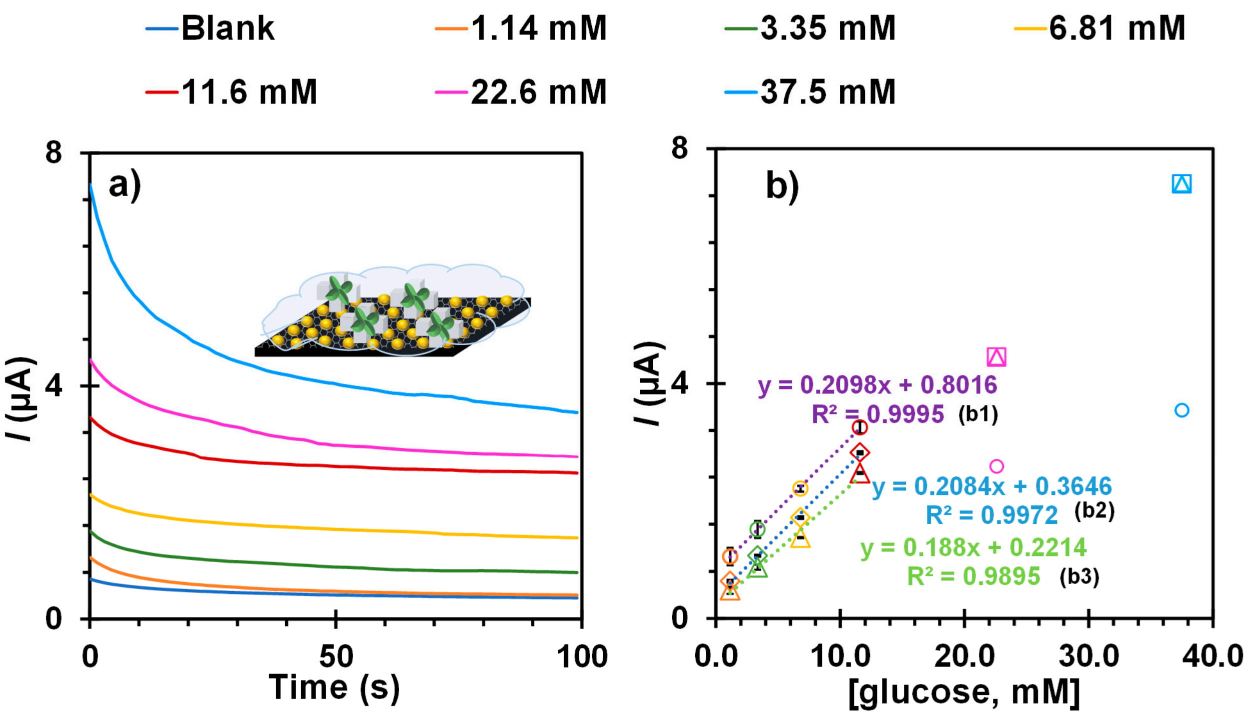

Figure S6: Chronoamperometric data (a) of direct readings of glucose on the C-SPEs/MIP-Py-COOH film with the corresponding representative calibration curves (b) in different time points: 0s (b1); 20s (b2) and 100s (b3).

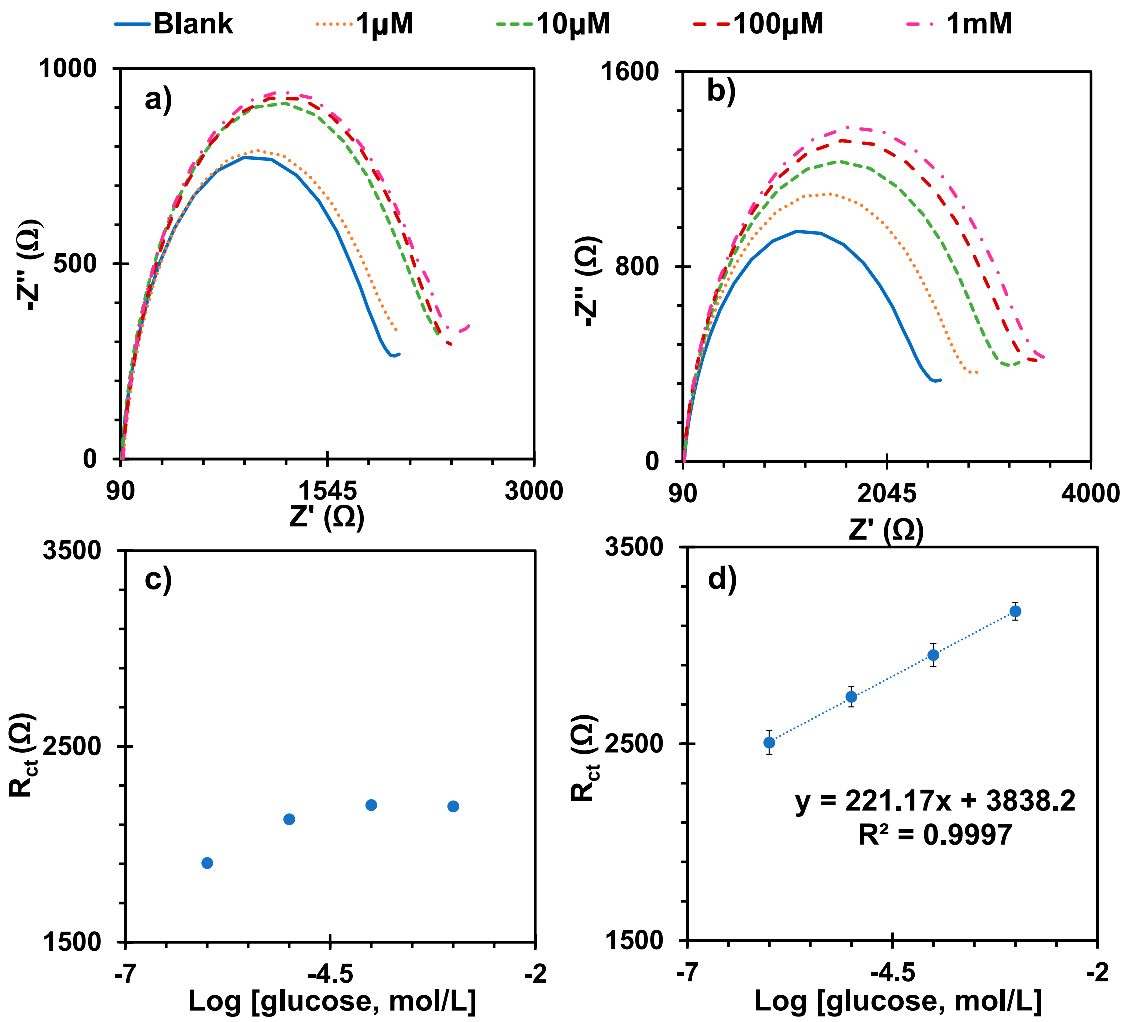

Figure S7: Calibration curves of the biosensors C-SPEs/AuNPs/MIP-NIP-Py-COOH, with (a) EIS measurements of NIP devices and (c) the corresponding calibration curves; (b) EIS measurements of MIP devices and the corresponding calibration curves (d) in serum 1000x diluted. Readings obtained in 5.0×10^{-3} M $[Fe(CN)_6]^{3-}$ and 5.0×10^{-3} M $[Fe(CN)_6]^{4-}$ solution prepared in PBS buffer, after incubation in increasing concentrations of glucose standard solutions.

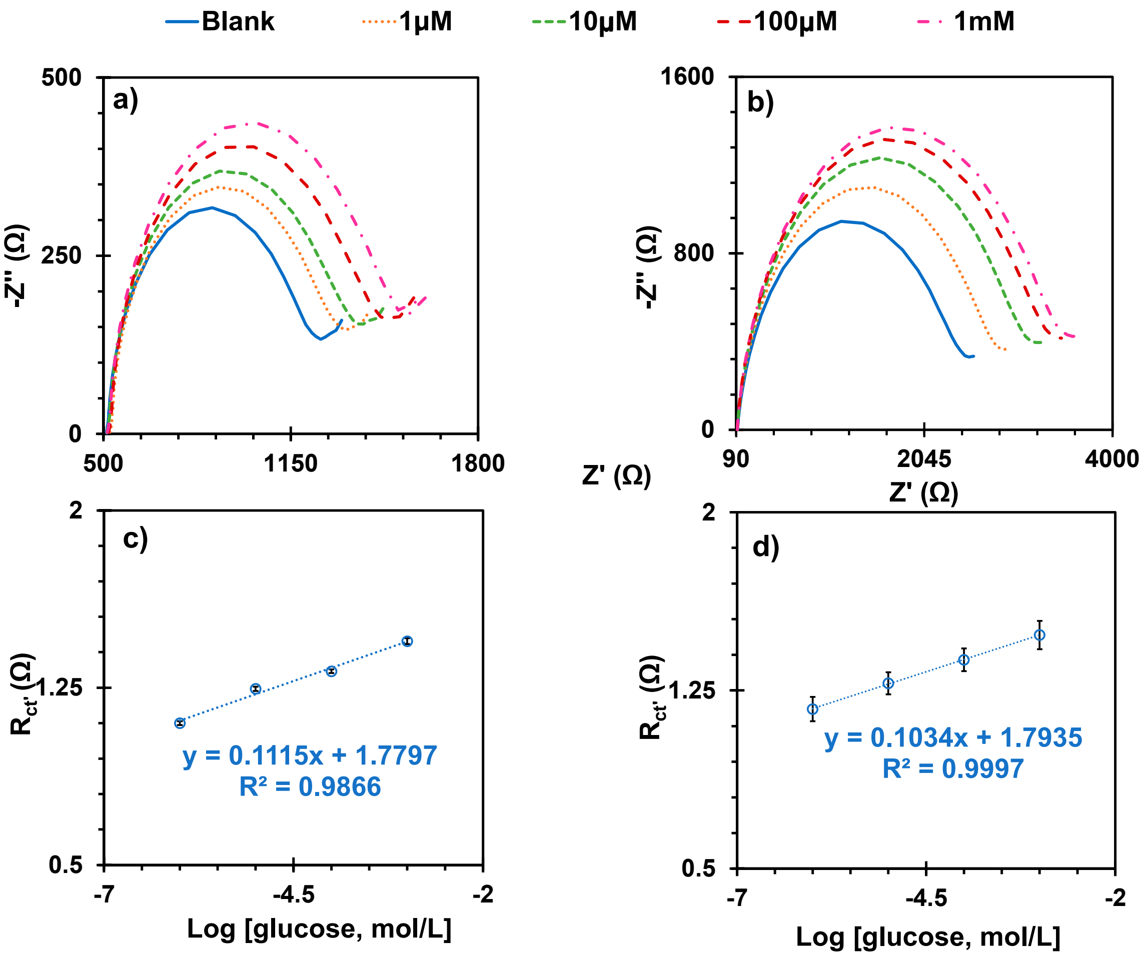

Figure S8: Calibration curves of the biosensors C-SPEs/AuNPs/MIP-NIP-Py-COOH, with EIS measurements in buffer (a) and serum (b) and the corresponding calibration curves with relative values (c) in buffer and (d) serum 1000x diluted. Readings obtained in 5.0×10^{-3} M $[Fe(CN)_6]^{3-}$ and 5.0×10^{-3} M $[Fe(CN)_6]^{4-}$ solution prepared in PBS buffer, after incubation in increasing concentrations of glucose standard solutions in different medium (buffer and serum).
